# Educational Intervention “Open Conversation and Reflection” to Enhance Person-Centeredness in Acute Geriatric Wards: A Qualitative Study of Consequences and Processes

**DOI:** 10.1089/pmr.2024.0080

**Published:** 2025-06-09

**Authors:** Karen Versluys, Sarah Janssens, Mieke Grypdonck, Let Dillen, Nele Van Den Noortgate, Linus Vanlaere, Ruth Piers

**Affiliations:** ^1^ Department of Geriatric Medicine, Ghent University Hospital, Ghent, Belgium.; ^2^ Howest University of Applied Sciences, Bruges, Belgium.; ^3^ Faculty of Medicine and Health Sciences, Department of Public Health, University Centre for Nursing & Midwifery, Ghent University, Ghent, Belgium.; ^4^ Department of Health Sciences, VIVES University College, Kortrijk, Belgium.; ^5^ Faculty of Theology and Religious Sciences, Research Unit Theological & Comparative Ethics, KULeuven, Leuven, Belgium.

**Keywords:** conversation, ethics, experiential learning, frail older adult, listening, person-centered care

## Abstract

**Background:**

Person-centered supportive care for older persons with acute illness is much needed but not easily achieved.

**Aims of the Study:**

To uncover processes and consequences of an educational intervention in acute geriatric wards intended as an exposure experience.

**Design:**

General inductive qualitative analysis was conducted on data from a four-step intervention: group coaching, an open conversation with a patient and family member (PT/FM), reflection on transcribed conversations with co-participant, and group peer reflection.

**Methods:**

Twelve participants engaged in the intervention. Transcripts from paired reflection sessions and peer reflections were analyzed using general inductive qualitative analysis.

**Results:**

The exposure experience of the participants involved three major processes: (1) fear before the conversation, (2) presence during the conversation, and (3) responsiveness after the conversation. Each process entailed several substeps. Not only the open conversation but also the whole process of reflection with a co-participant after reading the transcript and the peer-reflection sessions seem to be needed to realize the exposure experience.

**Conclusions:**

Through the educational intervention “open conversation and reflection,” participants experienced that connecting with PT/FM and listening to what is important to them are essential to realize person-centered care. Thus, pending further studies, the educational intervention can be considered promising to enhance person-centeredness in older people’s care.

## Introduction

Person-centered care—defined as care that aligns with a patient’s individual values and preferences—is critical but challenging, especially in managing frailty.^1–3^ Achieving person-centered care requires health care professionals (HCPs) to develop competencies that enable them to tailor care from the patient’s perspective.^4–8^ Baart’s theory of presence emphasizes exposure as a key—and for Baart even necessary—strategy to achieve perspective change.^9,10^ Exposure involves immersing oneself in the world of the patient to adopt the patient’s perspective and, in doing so, gains insight into patient’s life. This enables to align care more closely with the patient’s experiences and needs. Integration of a supportive, person-centered approach is needed as increasing numbers of frail older adults with acute health care needs are referred to hospitals.^11^ Notably, 26% of patients aged 75 years and older admitted to acute geriatric wards die within six months.^12,13^

Vanlaere et al. adapted Baart’s exposure methodology by developing simulation sessions where HCPs assumed the role of older patients.^14–16^ However, due to the time-intensive nature of simulations, an alternative method was explored and studied: the “open conversation and reflection” intervention. This educational intervention, structured in four steps, aims to provide HCPs with an exposure experience. This article describes the intervention and presents findings from a study that examined how participants experienced the exposure, whether they achieved perspective change and barriers and facilitators they identified.

## Methods

### The educational intervention

The “open conversation and reflection” program was developed and implemented in four stages over a three-month period. The first stage involved a group session where participants were introduced to the principles of person-centered care and coached on techniques for conducting attentive, open conversations. In the second stage, each participant, just prior to hospital discharge, engaged in an open conversation with a patient or a family member (PT/FM) of a patient they had cared for. The conversation focused on the PT/FM’s experiences during their hospital stay. These conversations took place at a location chosen by the PT/FM, scheduled outside of the HCP’s working hours. Participants were encouraged not to wear their uniforms, allowing them to step out of their professional roles and listen without constraints.

All conversations were audio-recorded and transcribed verbatim, enabling participants to read their own transcript and that of a co-participant in preparation for a reflective meeting in pairs. During this reflective meeting (stage 3), participants initiated a reflective dialogue with their co-participant using guiding questions to facilitate reflections on their experience (Table 1). Support from a researcher (K.V. and S.J.) was available if needed. The reflection aimed to help participants connect with their personal emotional responses to the open conversation rather than the clinical content of the patient’s care.

**Table 1.** Example Questions Used to Facilitate the Reflection in Pairs

How did you experience the conversation? / How was the conversation for you? What do you think about yourself as a partner in the conversation? At what point in the conversation did you succeed in encouraging the patient/family member to talk about their perceptions of being ill or their loved one’s illness? At what point in the conversation did you succeed in encouraging the patient/family member talk about their life? At what point in the conversation did you succeed in encouraging the patient/ family member talk about what is at stake for them? At what point in the conversation did you succeed in encouraging the patient/family member of a patient talk about their main concerns? What do you think contributed to your success in those moments?

The intervention concluded with a final reflective group session involving all participants (stage 4), led by the first author (K.V.) and observed by the second investigator (S.J.). This session focused on the participant’s overall exposure experience and its impact on themselves and their approach to care.

### Design

To answer the research question, a qualitative study was carried out. The educational intervention was delivered to a group of HCPs. Data generated by the educational intervention were used, supplemented by researchers’ observations and memos made during the process. The data were analyzed using a general inductive approach.^17,18^

### Participants and setting

The study was conducted in three acute wards of Ghent University Hospital, Belgium, specialized in the care of older adults admitted with frailty and acute medical conditions. These units are characterized by an interdisciplinary approach where team members collaboratively develop coordinated and integrated treatment and follow-up plans based on comprehensive geriatric assessment.^19^

The study’s objectives and procedures were first presented to the head nurses, who distributed the information to HCPs with a particular interest in person-centered care. The researchers subsequently contacted interested HCPs to confirm voluntary participation and provide further details about the study. Twelve HCPs (9 nurses and 3 occupational therapists) participated, including 2 males and 10 females, aged 23–51 years, all of Caucasian ethnicity. The intervention was facilitated by two investigators: a clinical nurse specialist in geriatric care (K.V.) and a clinical psychologist, specializing in care ethics (S.J.).

### Ethical considerations

The study protocol was approved by the Ethics Committee of the Ghent University Hospital, Belgium (registration number B670201524728). All participants, including PT/FMs and HCPs, received both written and verbal information about the study and provided informed consent to participate. All data were pseudonymized to ensure confidentiality. The preparation of this article followed the SQUIRE guidelines (Standards for Quality Improvement Reporting Excellence).^20^

### Data collection and analysis

Data generated during the intervention by the participants and used for analysis consist of the recorded and literally transcribed reflections in pairs and peer-reflection groups, supplemented with researchers’ observations (i.e., field notes taken during the reflections in pairs, where researchers [K.V. and S.J.] were present, as well as during the reflection groups^21^ and memos created throughout the interpretation process). The analysis began with a close reading of the data to form initial insights into their meaning. All obtained data were analyzed, inductively coded (without using a predetermined coding scheme) and analyzed as a whole.^22,23^ An interdisciplinary team of researchers (K.V., S.J., M.G.), with expertise in geriatric care, qualitative research, and health care education, participated in all stages of the intervention design and data analysis (investigator triangulation). Emerging themes were identified through constant comparative analysis, refining a model of core concepts, while maintaining each transcript’s unique aspects. Both within-case and across-case analysis ensured thorough connections between categories.

The interpretative process, guided by the critical friends approach,^24^ was grounded in reflexivity to ensure openness to the data’s meaning and to foster a continual questioning of the interpretations.^25^ The final concepts were entered into NVivo 11 (QSR International) for analysis. Finally, the resulting framework was reviewed with an advisory panel (L.D., L.V., N.V.D.N., and R.P.).

## Findings

### Participants

Ten participants conducted one conversation each, while two conducted conversations with both a patient and a family member, resulting in 14 conversations. Conversation durations ranged from 13 to 64 minutes, with an average of 32 minutes. All participants completed the intervention’s four stages.

While participants could request facilitation from researchers (K.V. and S.J.) during reflection in pairs, only one of seven pairs sought this support. Reflections in pairs lasted between 25 and 86 minutes, with an average of 53 minutes. The final reflective group session lasted 120 minutes.

### Process of exposure

**FIG. 1.**
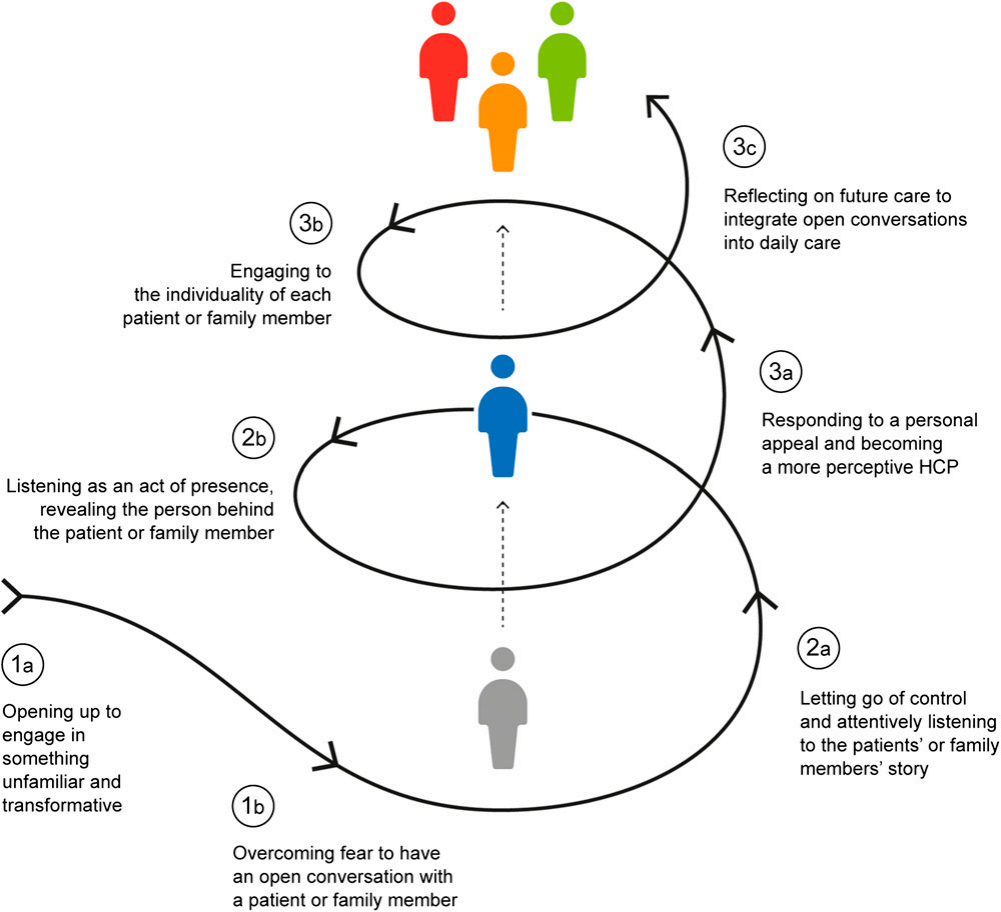
This framework outlines the process of the participant’s exposure experience in the educational intervention “open conversation and reflection”: before (1a–b), during (2a–b), and after (3a–c) the conversation. Through listening and presence, participants began to perceive the care receiver as a person. Subsequently, reflections highlighted the awareness that connecting with and listening to what is important to each care receiver is a fundamental step in care.

The study uncovered the process participants in the educational intervention went through to achieve a perspective change that, as they said, was an important changemaker for their practice. Figure 1 illustrates this process, which unfolds across three themes: fear before, presence during, and responsiveness after the conversation. Each theme includes subthemes that provide deeper insights.

1. Fear before the conversation
  a. **Opening up to engage in something unfamiliar and transformative**
    Following the group session (stage 1), participants felt encouraged to initiate an open conversation with a PT/FM. This required them to step outside their comfort zone and embrace the inherent uncertainty of the encounter. Participants reported feelings of uncertainty and stress about the emotional and relational outcomes of such conversation. They feared the unknown and were concerned that the conversation might elicit emotions in the PT/FM (Table 2, Q1).

**Table 2.** Quotes

	The process of exposure
1. Fear before the conversation	
Q1	(…) patients getting emotional: I’m afraid of that. It is already difficult for patients to be admitted to a hospital and then we start a conversation that makes them feel emotional. (Female nurse—44 years, patient, reflection in pairs)
Q2	I was too busy hoping to have a good conversation. I wanted to hear everything about the patient’s feelings. This had the opposite effect. (Female nurse—35 years, patient, reflection in pairs)
Q3	That gave me quite some stress! The idea that they [=the researchers] will listen to this conversation. “Am I doing it right?’ Someone will transcribe this and others will read this (…). Yes, I was constantly thinking: I must succeed in this conversation.” (Female occupational therapist—28 years, family member, reflective group session)
2. Presence during the conversation	
Q4	I was very much preoccupied with the questions I wanted to ask. I thought: “I can’t ask that” or “what will be my next question?” (…) “and then, I realized, I don’t have to think about what I need to ask, … I just have to let him talk and I must try to hear what he wants to tell me. (Female nurse—35 years, patient, reflection in pairs)
Q5	I wouldn’t do it [=talking about personal experiences and emotions] either (…) You can’t just expose yourself to someone you’ve never seen before. (Female occupational therapist—28 years, family, reflection in pairs)
Q6	I had a conversation with a patient I already knew for a long time. And I was very surprised. The conversation touched me deeply. The whole time she was in the hospital, she never told me such things. I’d never thought she would tell all this. Quite unexpectedly, she told me so much, even emotional things. I was really astonished. (…) She told me so many things, I would never have learned about without the conversation. I am ashamed actually. Just ashamed, that we didn’t hear this information during our usual care. (Female nurse—31 years, patient, reflection in pairs)
3. Responsiveness after the conversation	
Q7	(…) In 20 minutes she gave me so much information of who she is: a strong woman who always took care for her children and family, … a proud woman who now has difficulties with having an old, declining body and who has nobody left to take care for. Now she needs care. And that was very hard for her. (…) Now I understand why she was so resisting during her stay in the hospital: she felt so dependent. This is very important to take into account in our care and therapy. (Female occupational therapist—41 years, patient, reflection in pairs)
Q8	Afterwards I thought: “Oh, my God, what a conversation was this!” I really had to go outside to think over what the patient had told me and all the misery she had. (…) I really learned that there can be lots of pain behind a cheerful face. You can’t really know a person just by seeing him. (Female nurse—23 years, patient, reflective group session)
Q9	“For us, caregivers, these things are natural because we have done this frequently. Now, I realize, they are not so natural for patients. (…) It is hard to lay in bed and not being able to do anything, being dependent of others who just run out on the moment you want to ask something.(…) I’m much more aware that I have to stand still with the patient and with what I’m doing.” (…) Now, I’m much more attentive to what the patient says and how he feels in that situation, how the relation is with his children, … (Female nurse—35 years, patient and family, reflective group session)
Q10	I think I really needed this conversation so that my future conversation would be in the right direction. Yes, I’ve had stress and I asked myself 10 times “why did I start this?”. The feeling, the satisfaction afterwards is much bigger because of the fact that the impediment was so high. I needed to take this hurdle. (Female nurse—35 years, patient, reflective group session)

  b. **Overcoming fear**
    Participants reported feeling tense about engaging in this new experience, recognizing the need to overcome a certain level of fear to fully engage in active listening. Several factors contributed to this fear. First, participant’s self-criticism created internal pressure. Despite reassurances during the group session that attentive listening was sufficient, participants remained afraid of failing to facilitate a meaningful conversation. Some participants set personal goals, aiming for an encounter that would be both meaningful for the PT/FM and successful for themselves (Table 2, Q2).
    Second, some participants expressed concerns about being judged, as their conversation was recorded and reviewed by researchers and co-participants (Table 2, Q3).
2. Presence during the conversation
  a. **Letting go of control and engaging with the patient’s story**
    As the conversation progressed, most participants overcame their fears and relinquished control over the dialogue. By releasing this control, they became more open and created a deeper connection with the PT/FM. From this point onward, the conversation shifted from small talk to a more meaningful, in-depth encounter centered on the PT/FM’s experiences (Table 2, Q4).
    However, a few participants struggled to relinquish their preoccupations. These individuals perceived the open conversation as a threatening disclosure of themselves within a professional relationship (Table 2, Q5). As a result, they were unable to achieve the openness required to truly listen to the PT/FM.
  b. **The conversation as an act of presence, revealing the person behind the PT/FM**
    Participants who were able to create space for the PT/FM gained the insight that truly listening and being present as a person involve more than simply holding a conversation. Through the conversation, participants began to perceive the PT/FM as a person, revealing the individual behind the role of PT/FM. Consequently, they gained a clearer understanding of the PT/FM’s experiential world. What the PT/FM shared deeply moved the participants (Table 2, Q6), and this emotional resonance helped them recognize the shared humanity between themselves and the PT/FM. By acknowledging the PT/FM’s vulnerability and the asymmetry in the care relationship, participants reflected on how an open dialogue could foster mutual understanding.
3. Responsiveness after the conversation
  a. **Responding to a personal appeal and becoming a more perceptive HCP**
    Through their attunement to the PT/FM, participants gained clarity about what truly mattered to the PT/FM. After the conversation, the HCP’s perspective shifted: rather than being focused solely on tasks, they began to explore the PT/FM’s story more deeply through genuine, meaningful dialogue (Table 2, Q7). This transformation became especially evident after they reviewed the transcripts of their conversations.
    While reading and reflecting, participants felt themselves immersed in the PT/FM’s full narrative. This reflections heightened their awareness of the emotional dimensions of the PT/FM’s experience. Many reported feelings as though they had, in some way, stepped into the PT/FM shoes. This experience inspired them to approach caregiving with greater compassion and responsiveness than before (Table 2, Q8).
  b. **Attuning to PT/FM’s lived experience**
    Participants discovered that connecting with the PT/FM and listening to what is important to them is a fundamental step in delivering person-centered care. By sharing their experiences in the reflection group, participants reflected on the core principles of care, their own competencies and behaviors, and their intentions regarding future practice. They acknowledged that their previous approach to care thus far had been focused on problem-solving, assuming that achieving professional goals was the most effective course of action. Now they realized that this task-oriented approach had caused them to overlook the PT/FM’s personal experiences. Most participants concluded that engaging in such conversations was not only beneficial to their caregiving, but also personally rewarding. They became more aware of the importance of care rooted in the PT/FM’s perspective and the mutual relationship between HCP and PT/FM. (Table 2, Q9).
  c. **Reflecting on future care**
    During the reflection group, participants highlighted several contextual factors necessary to integrate open conversations into daily care. While they initially struggled to discuss the PT/FM’s experiential world, they ultimately found the process rewarding. By listening to and understanding what truly mattered to the PT/FM, they found deeper meaning to their roles as HCPs and felt motivated to pursue person-centered care more actively (Table 2, Q10).

### Conditions facilitating the process of exposure

During the process of reflections in pairs and in group, participants reflected on what supported their perspective change and what they considered necessary for creating an impact within their team. In what follows, we report conditions participants identified as crucial for facilitating the exposure process and its influence on person-centered care.

1. Conditions for fruitful conversations with PT/FMs
  Sufficient time for the conversation, scheduled during off-duty hours, helped reducing pressure on participants. Additionally, the choice of location and ensuring privacy also played a role in its success (Table 3, Q11).
  Some participants felt that audio recording hindered the authenticity of the interaction. Addressing this issue, such as pausing the recording when needed, resulted in more genuine conversations (Table 3, Q12).
2. Conditions for reflection
  Carefully reviewing the conversation transcript was a critical step in preparing for the reflection process (Table 3, Q13). A few participants who were less committed and did not thoroughly review the transcript in advance struggled to engage in deep reflection (Table 3, Q14). They could not fully engage with their own lived experiences during the reflection and instead followed the contributions of others in the group.
  However, reflecting in pairs provided a safe space for participants to explore the significance of the open conversation. These initial reflections were essential in helping them realize that letting go of their own preoccupations and genuinely listening to the PT/FM was the right approach.
3. Conditions for application in practice
  In the reflective group session, participants acknowledged the value of their shared exposure experience. They gained a deeper understanding of each other and realized they shared a common vision of person-centered care (Table 3, Q15).
  However, when working together with colleagues who had not attended the intervention, participants faced obstacles in applying their insights, such as team culture, lack of understanding or motivation among colleagues, collegial judgments, high work pressure, and procedural constraints (Table 3, Q16–Q17).
  Many participants expressed a desire for all team members to undergo a similar exposure experience and perspective shift and to undertake this collectively. They indicated the need for support and alignment within the team, emphasizing that if left alone with these experiences, they would be unable to achieve the person-centeredness they now understand is possible and needed.

**Table 3.** Conditions Facilitating the Process of Exposure

1. Conditions for fruitful conversations with patient or family member	
Q11	Suddenly another colleague and her student came in the room and sat down to listen. That was really annoying. The patient kept on telling her story. But for me, that was stressful and distracting. (…) You are talking about something very personal and when someone interrupts the conversation, you suddenly loose that moment of trust… (Male nurse—25 years, patient, reflection in pairs)
Q12	‘At a certain moment she [=patient] said very directly: ‘If that [=audio-recorder] wouldn’t be here, there wouldn’t be a problem. When I stopped recording, she needed some time to recover.’ (Female nurse — 47 years, patient, reflection in pairs)
2. Conditions for reflection	
Q13	Reading the transcripts of the conversations is worthwhile. After the conversation, it seemed I couldn’t remember exactly what the patient and I really talked about. I was surprised reading the whole conversation. (Female Occupational therapist—41 years, patient and family, reflection in pairs)
Q14	… I just read the transcript quickly. I didn’t read it with my attention. I’d better done this. . . (Female nurse—38 years, patient, reflection in pairs)
3. Conditions for application in practice	
Q15	During the program, you learn to know and to appreciate each other. You learn what’s important to your colleagues. The program creates a certain connection and that is good. (Female occupational therapist—41 years, patient and family, reflection in pairs)
Q16	Sometimes I miss a colleague to share these experiences with. I hear a lot from patients that really touches me. Sometimes I tell this to the nurses and then they look at me in a way “what is going on with you?” … That touches me, it makes me sad. (…) Sometimes I have the feeling that colleagues don’t understand why I do my job, the way I’m doing it. They think I’m busy for a long time with one patient. But it is my job to do that. (Female occupational therapist—41 years, patient and family, reflective group session)
Q17	(…) I wanted to take time and let the patient go deeper in his emotions, but I was worried because I had already spent quite some time. (…) there are other patients waiting. (Female occupational therapist—28 years, family, reflection in pairs)

## Discussion

Person-centered care is a prominent topic in contemporary health care, particularly in the care of vulnerable older adults. Understanding the patient’s experiential world and HCP’s responsiveness is essential for achieving person-centered care. This study explored an educational intervention designed to enable HCPs to practice a perspective shift. Based on participants’ reflections and actions, one can infer that the intervention achieved its goal. To our knowledge, this is one of the first studies to explore reflective practice as a means of exposure in bedside care for frail older patients through open conversation.

Findings suggest that attentive listening allowed most participants to immerse themselves in the PT/FM’s world, becoming profoundly moved by their stories. The perspective shift enabled participants to adopt the PT/FM’s viewpoint, giving deeper meaning to their role as HCP and motivating them to pursue genuine person-centered care. Participants did more than simply listen; they listened with “engaged awareness”—being attentive, present in-the-moment, and emotionally and empathically involved—while embodying “person-centeredness,” as described by King.^26^ Baart similarly emphasized that this perspective shift, achieved through active listening, is a key element in the exposure experience, leading to deeper understanding of the PT/FM.^12^ Gallagher referred to this shift as “epiphanous insights with a powerful immediate impact,”^27^ enhancing HCPs’ awareness of their ethical intuitions and perceptions.^14^

The exposure experience unfolded progressively throughout the intervention. Initially, some participants perceived the idea of an open conversation, as a barrier. However, most eventually overcame their concerns and listened to the PT/FM in an open and attentive manner, although a few continued to struggle. Devenny and Duffy similarly identified self-awareness as a significant challenge for HCPs in comparable programs.^8^ They emphasized the value of reviewing conversation transcripts and reflecting on personal experiences. In their ETHoS-project, Devenny and Duffy employed a reflective approach that allowed participants to explore how their values and beliefs are incorporated into clinical practice.^8^ Our intervention extended beyond personal awareness of one’s own feelings, values and beliefs to achieving attunement in care. The subsequent reflection sessions fostered an adjusted and shared vision of person-centered care among participants.

Participants in our study sought to personalize care using insights from the exposure experience but faced obstacles such as traditional approaches and poor communication. They expressed the need for a “shared understanding” among colleagues and wished for team-wide participation in the intervention. Achieving a shift in care vision, where the entire team values and rewards meaningful engagement with PT/FMs, is essential for realizing person-centered care.^4,28^

This study underscores the potential of open conversations in enabling HCPs understand PT/FM’s perspective and collaborate with them to build relationships that inform and support decision making. Genuine listening and eliciting the PT/FM’s story require specific competencies.^29^ Given the complexity and centrality of authentic listening for care outcomes, investing in learning communities where HCPs can develop and refine skills as “engaged and person-centered listeners” seems crucial.^26,30^

Several projects, such as “my life, my story” intervention, use patient narratives to integrate the patient’s life story into care, preventing the decontextualization of the patient.^31^ In contrast, our intervention centers on PT/FM’s experiences during hospitalization. Reflection on one’s own practice based on the patient’s story is key to the exposure methodology. Which of the two approaches is stronger in leading to the goal of person-centered care will need further investigation.

### Strengths and limitations

The educational intervention represents an innovative approach to fostering reflexivity in practice, grounded in listening, as participants arrived at similar insights.

The perspective shift observed may be attributed to the voluntary nature of participation, as those who volunteered may have already had a sense of person-centeredness in their care. Future research should assess the program’s impact on PT/FMs and explore its effectiveness when used by an entire team.

The study mainly made use of the data generated during the educational process. Testing emerging insights with directed questions would have interfered. Further systematic investigation of the intervention is needed to strengthen the conclusions of this study.

## Conclusions

This article describes a short educational intervention where participants experienced exposure through open conversations with a PT/FM, followed by peer reflection. This exposure experience led to a perspective shift allowing participants to view care through the PT/FM’s perspective. Key elements leading to the exposure experience included the open conversation itself, the transcript review, and subsequent peer reflection in pairs and group. While this intervention can support HCPs in achieving genuine person-centered care, further efforts are needed to foster a shared vision within the interdisciplinary team.

## Data Availability

All data relevant to the study can be attained through reasonable request from the principal investigator.

## Abbreviations Used

HCP: health care professional
PT/FM: patient and family member

## References

[1] Nicholson C, Morrow EM, Hicks A, et al. Supportive care for older people with frailty in hospital: An integrative review. Int J Nurs Stud 2017;66:60–71; doi: 10.1016/j.ijnurstu.2016.11.015 28012311

[2] De Raedt S, De Groote M, Martens H, et al. Will-to-live and self-rated health in older hospitalized patients are not predictive for short-term mortality. J Palliat Med 2024;27(3):376–382; doi: 10.1089/jpm.2023.0326 37948556

[3] Piers R, De Brauwer I, Baeyens H, et al. Supportive and palliative care indicators toll prognostic value in older hospitalised patients: A prospective multicentre study. BMJ Support Palliat Care 2021;14(e2):1–8; doi: 10.1136/bmjspcare-2021-003042 PMC11672032 34059507

[4] The American Geriatrics Society Expert Panel on Person-Centered Care. Person-centered care: A definition and essential elements. J Am Geriatr Soc 2016;64(1):15–18; doi: 10.1111/jgs.13866 26626262

[5] Dewar B, Nolan M. Caring about caring: Developing a model to implement compassionate relationship centred care in an older people care setting. Int J Nurs Stud 2013;50(9):1247–1258; doi: 10.1016/j.ijnurstu.2013.01.008 23427893

[6] Lynn J, McKethan A, Jha AK. Value-based payments require valuing what matters to patients. JAMA 2015;314(14):1445–1446; doi: 10.1001/jama.2015.8909 26378889

[7] Moore L, Britten N, Lydahl D, et al. Barriers and facilitators to the implementation of person-centred care in different healthcare contexts. Scand J Caring Sci 2017;31(4):662–673; doi: 10.1111/scs.12376 27859459 PMC5724704

[8] Devenny B, Duffy K. Person-centred reflective practice. Nurs Stand 2014 Mar 12-18;28(28):37–43; doi: 10.7748/ns2014.03.28.28.37.e8068 24617403

[9] Price A. Encouraging reflection and critical thinking in practice. Nurs Stand 2004;18(47):46–52; doi: 10.7748/ns2004.08.18.47.46.c3664 15357553

[10] Caldwell L, Grobbel C. The importance of reflective practice in nursing. Int J Caring Sci 2013;6(3):319–326.

[11] Vanlaere L, Gastmans C. Ethics in nursing education: Learning to reflect on care practices. Nurs Ethics 2007;14(6):758–766; doi: 10.1177/0969733007082116 17901186

[12] Baart A, Grypdonck M. Nursing and Presence – A Dialogue Search for the Meaning of Presence for Nursing Care. 1st ed. Lemma: Utrecht; 2008.

[13] Baart A. A Theory of Presence. 2nd ed. Lemma: Utrecht; 2001.

[14] Vanlaere L, Coucke T, Gastmans C. Experiential learning of empathy in a care-ethics lab. Nurs Ethics 2010;17(3):325–336; doi: 10.1177/0969733010361440 20444774

[15] Vanlaere L, Timmermann M, Stevens M, et al. An explorative study of experiences of healthcare providers posing as simulated care receivers in a ‘care-ethical’ lab. Nurs Ethics 2012;19(1):68–79; doi: 10.1177/0969733011412103 22140188

[16] Vanlaere L, Burggraeve R. Bodily contrast experiences in cultivating character for care. Nurs Ethics 2024;31(1):7–16; doi: 10.1177/09697330231171709 37200623

[17] Creswell J. Qualitative methods. In: Research Design: Qualitative, Quantitative and Mixed Methods Approaches. Sage: London; 2022.

[18] Thomas D. A general inductive approach for analyzing qualitative evaluation data. Am J Eval 2006;27(2):237–246; doi: 10.1177/1098214005283748

[19] O’Shaughnessy Í, Robinson K, O’Connor M, et al. Effectiveness of acute geriatric unit care on functional decline, clinical and process outcomes among hospitalized older adults with acute medical complaints: A systematic review and meta-analysis. Age Ageing 2022;51(4):1–11; doi: 10.1093/ageing/afac081 PMC9053463 35486670

[20] Ogrinc G, Davies L, Goodman D, et al. SQUIRE 2.0 (Standards for Quality Improvement Reporting Excellence): Revised publication guidelines from a detailed consensus process. BMJ Qual Saf 2016;25(12):986–992; doi: 10.1136/bmjqs-2015-004411 PMC5256233 26369893

[21] Tong A, Sainsbury P, Craig J. Consolidated criteria for reporting qualitative research (COREQ): A 32-item checklist for interviews and focus groups. Int J Qual Health Care 2007;19(6):349–357; doi: 10.1093/intqhc/mzm042 17872937

[22] Sebastian K. Distinguishing between the types of grounded theory: Classical, interpretative and constructivist. Jst 2019;3(1):1–9.

[23] Higginbottom G, Lauridsen EI. The roots and development of constructivist grounded theory. Nurse Res 2014;21(5):8–13; doi: 10.7748/nr.21.5.8.e1208 24877905

[24] Bambino D. Critical Friends. Educational Leadership 2002;59(6):25–27.

[25] Malterud K. Qualitative research: Standards, challenges, and guidelines. Lancet 2001;358(9280):483–488; doi: 10.1016/S0140-6736(01)05627-6 11513933

[26] King G. Central yet overlooked: Engaged and person-centred listening in rehabilitation and healthcare conversations. Disabil Rehabil 2022;44(24):7664–7676; doi: 10.1080/09638288.2021.1982026 34647516

[27] Gallagher A, Peacock M, Zasada M, et al. Care-givers’ reflections on an ethics education immersive simulation care experience: A series of epiphanous events. Nurs Inq 2017;24(3):1–10; doi: 10.1111/nin.12174 28004462

[28] Nolan MR, Davies S, Brown J, et al. Beyond ‘person-centred’ care: A new vision for gerontological nursing. J Clin Nurs 2004;13(s1):45–53; doi: 10.1111/j.1365-2702.2004.00926.x 15028039

[29] Naldemirci O, Britten N, Lloyd H, et al. The potential and pitfalls of narrative elicitation in person-centred care. Health Expect 2020;23(1):238–246; doi: 10.1111/hex.12998 31743559 PMC6978872

[30] Timmerman G, Baart A. The continuing formation of relational caring professionals. Med Health Care Philos 2022;25(4):587–602; doi: 10.1007/s11019-022-10104-0 36029426 PMC9613723

[31] Nathan S, Fiore LL, Saunders S, et al. My life, my story: Teaching patient centered care competencies for older adults through life story work. Gerontol Geriatr Educ 2022;43(2):225–238; doi: 10.1080/02701960.2019.1665038 31498034

